# Editorial Decisions May Perpetuate Belief in Invalid Research Findings

**DOI:** 10.1371/journal.pone.0073364

**Published:** 2013-09-04

**Authors:** Kimmo Eriksson, Brent Simpson

**Affiliations:** 1 School of Education, Culture and Communication, Mälardalen University, Västerås, Sweden; 2 Centre for the Study of Cultural Evolution, Stockholm University, Stockholm, Sweden; 3 Department of Sociology, University of South Carolina, Columbia, South Carolina, United States of America; Université de Montréal, Canada

## Abstract

Social psychology and related disciplines are seeing a resurgence of interest in replication, as well as actual replication efforts. But prior work suggests that even a clear demonstration that a finding is invalid often fails to shake acceptance of the finding. This threatens the full impact of these replication efforts. Here we show that the actions of two key players – journal editors and the authors of original (invalidated) research findings – are critical to the broader public’s continued belief in an invalidated research conclusion. Across three experiments, we show that belief in an invalidated finding falls sharply when a critical failed replication is published in the same – versus different – journal as the original finding, and when the authors of the original finding acknowledge that the new findings invalidate their conclusions. We conclude by discussing policy implications of our key findings.

## Introduction

Social psychologists agree that replication is essential to a healthy science, yet actual replication efforts have been extremely rare. This is largely because disciplinary incentive systems have tended to ghettoize replication efforts, relegating them to lower ranked journals or not publishing them at all [1], [2]. This is part of a more general trend in which the publication of negative results has become increasingly rare in many disciplines, especially the social sciences [3], [4]. But a new crisis in social psychology, stemming from widely publicized issues of “fraud, replication, publication bias, and false positive results” [5] has led to a renewed interest in replication. Indeed, there are now ambitious efforts, perhaps most notably The Reproducability Project led by Brian Nosek [6], to assess the reproducibility of findings in social psychology. If continued, widespread replication efforts should yield more realistic estimates of what portion of social psychological findings are real and robust and thus give us a better of sense of what we do and do not know about human behavior and psychology.

But here we address a second hurdle facing replication efforts, especially those that disconfirm prior findings. Even with strong contradictory evidence in hand, original findings often continue to live on, in support of “undead theories” [7]. We suggest that this is especially likely for the types of theories and research findings most apt to be published in high impact journals and widely cited in social psychology: those that are counter-intuitive or otherwise “sticky” [8]. (Invalidated findings might also outlive their evidentiary basis because their implications are more benign than reality, as Nolan [9] notes in his review of discredited findings that persist in sociology: “Interestingly, in many cases when these fantastic claims were shown to be false, their untruth was considered by many to be less important than the good that resulted from the increased attention and the efforts at remediation that their trumpeting in the media produced.” An example from social psychology is the popular but discredited story of 38 witnesses who failed to act in the Kitty Genovese murder [10], [11].) If no mechanism exists to ensure that disconfirming evidence is widely shared and accepted, then the full potential of emerging replication efforts will likely go unrealized.

How can claims that do not withstand further empirical scrutiny be prevented from becoming “glorified anecdotes” (Nisbett, quoted in [12]) that continue to be treated as fact, despite the existence of compelling contradictory evidence? Or, more specifically, what determines the chance that a corrective finding will reach public awareness? Here we focus on the role of two key players in the scientific process: journal editors and the authors of the original, discredited, finding.

We focus on whether an editor decides to publish a failure to replicate because, in contrast to journals in the physical sciences [13], major journals in social psychology will decline to publish replications as a matter of policy. This was recently illustrated in the aftermath of the publication of a paper on precognition in *Journal of Personality and Social Psychology*, as reported by Aldhous [14]. We argue that an editor’s decision not to publish contradictory evidence gives credence to the original, disproven finding, even if the contradictory evidence is published elsewhere. If so, editorial decisions or policies not to publish failures to replicate may help explain the persistence of undead theories in social psychology and related disciplines.

Likewise we argue that responses of the authors of the original findings are important to determining whether a failure to replicate takes hold in the mind of the scientific community or the broader public. The original authors may not respond at all, which can keep the failure to replicate from attracting much attention. Failures to replicate might also result in “debates” characterized by “sweeping dismissals and oblique deflections” [15] or that otherwise fail to meet the standards of accuracy for publication in peer-reviewed journals. (For unclear reasons, it seems these debates are often not peer-reviewed like other articles.) Authors of the original finding may, for instance, claim that the failure to replicate was caused by inexperienced scientists who did not follow appropriate procedures. (For a recent example of this kind of dismissal of failed replications, see the debate on the validity of some behavioral priming experiments [16].) Readers may not know how to properly judge these competing claims and therefore give less credence to the contradictory evidence than is warranted. As a result, we argue, how the authors of the original finding respond is key to whether the public continues to put faith in invalidated findings.

We tested our arguments in three studies. As detailed below, all three studies show that whether or not a discredited finding continues to be accepted as true depends on the actions of the editor of the journal in which the original finding was published. The first two studies used a 2-by-2 within-subjects design, manipulating both the journal in which the contradictory evidence appeared (the same journal in which the original finding was published, or a different journal) and the original authors’ reaction (whether they acknowledge the contradictory evidence as invalidating their finding). Participants in these two studies were selected from a convenience sample. Study 3 was designed to address several weaknesses in the first two studies by using a between-subjects design and employing participants with advanced degrees in social science.

As evident in our earlier discussion, an attempt to replicate a prior finding using the same procedures and methods as the original study may fail to find the same effects for several different reasons. A replication attempt could “fail” because the original finding was not real or reliable. Alternatively, an attempt to replicate a real or reliable finding could fail because the replication researchers do not implement some critical part of the design, or simply due to chance. We are most interested in the role of journal editors and original authors in cases where the underlying reason for the contradictory evidence is not so ambiguous [17]. In Studies 1 and 2, we simply used the term “contradictory evidence,” but in the third study we explicitly described the evidence in terms of a stronger form of replication study that critically explains the original findings as being based on an artifact of the study design. In this type of “critical failed replication” the follow-up researchers first replicate the original result and then show that it fails to replicate when the source of the artifact is modified.

## Studies

All three studies were conducted by the first author as anonymous online questionnaires with users of Amazon Mechanical Turk (hereafter AMT; mturk.com).

### Ethics Statement

No approval was obtained. For research conducted in Sweden, need for approval is regulated by the Act concerning the Ethical Review of Research Involving Humans (2003:460), which can be accessed in official English translation at the web site of the Central Ethical Review Board (www.epn.se). What research needs approval is described in Sections 3 and 4 of the Act. In brief, studies with human participants need approval only if they involve sensitive personal data (defined as race or ethnic origin, political opinions, religious or philosophical beliefs, or membership of a trade union, and data on health or sex life) or use a method intended to physically or mentally influence a person. Our studies clearly do not involve sensitive personal data according to the definition. Further, the method of having people fill in questionnaires or make decisions do not count as intended to physically or mentally influence a person, as confirmed by a previous application by the first author to the Regional Ethical Review Board in Uppsala, Sweden.

Participants were clearly informed that by submitting their responses to the questionnaire they consented to the responses being used for research.

## Study 1

Our first study investigated how belief in a published research finding is affected by contradictory evidence, depending on whether it is published in the same journal or in another, lower-ranked, journal, and whether the authors of the original study acknowledge the contradictory evidence or remain silent.

### Participants

Ninety-six American users of AMT (34% female; mean age 34 years), of varied educational background, completed a survey for a compensation of $0.50.

### Method

Participants were asked to consider the following scenario: “Through a very credible video, widely shared on Twitter and Facebook, you learn of a remarkable finding published in a prestigious journal by a group of social scientists. The finding rings true with you, and you share it with your friends who are equally enthusiastic. When surfing the internet, you incidentally find mention of a report where some unknown researchers claim that the original finding does not hold.”

In a 2-by-2 within subjects design, participants were asked to consider four different ways in which a journal editor and the authors of the original finding responded to the contradictory evidence: First, they were either told that the report was published in *the same prestigious journal* as the original finding, or that it was published in *a low-ranked online journal.* The order of these two scenarios was counterbalanced across participants. Thereafter, participants read another two scenarios, which were identical to the previous ones up to the end, at which point the same piece of information was added to both scenarios: “The report also mentions that the researchers who reported the initial finding acknowledge that this new report indeed invalidates their original finding.” For each of the four scenarios, participants were simply asked to rate the likelihood (from 0% to 100%) that the original finding was correct or valid.

### Results

**Figure 1:**
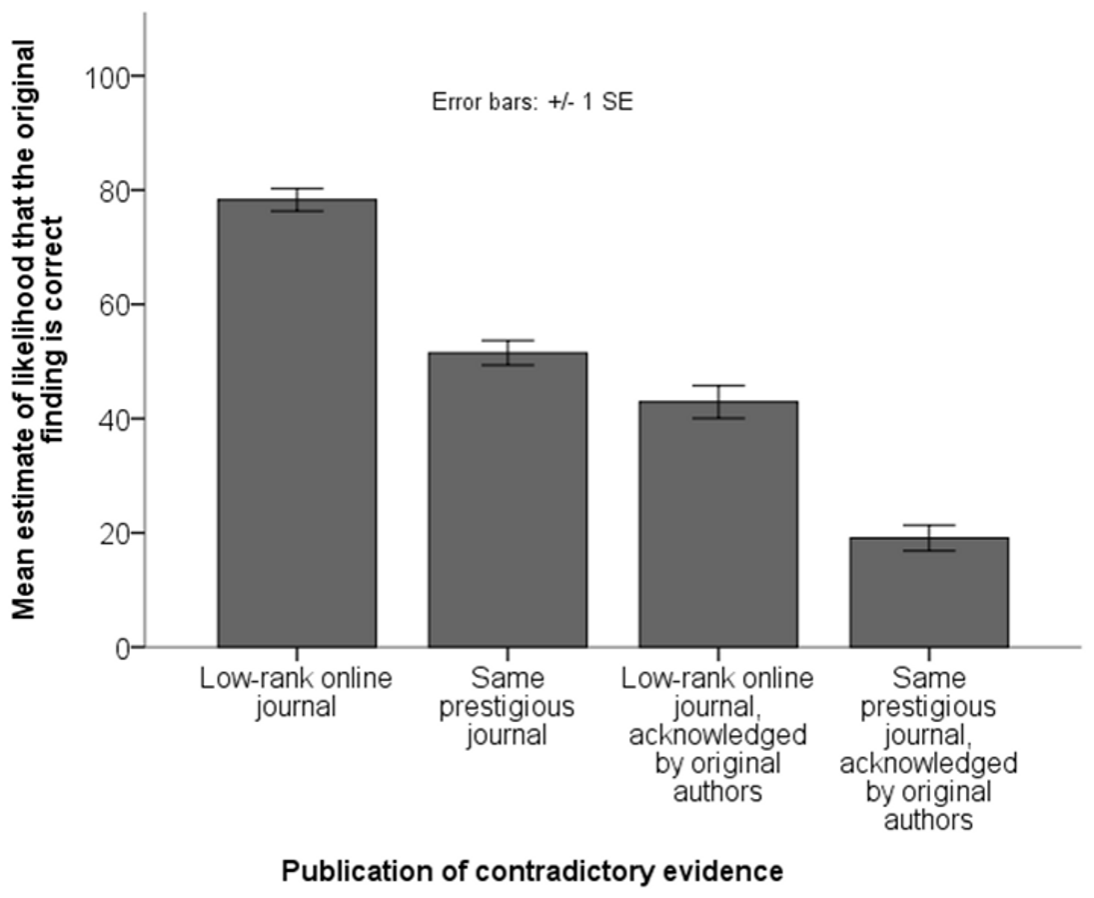
Results from Study 1. Belief in an original finding, for which subsequent contradictory evidence exists, depends on whether the contradictory evidence is published in the same prestigious journal as the original finding and whether the original authors acknowledge the contradictory evidence.

Figure 1 shows that, when the contradictory evidence was neither published in the same journal, nor acknowledged by the original authors, belief in the original finding was quite high (*M* = 78.9, *SD* = 18.9). Belief in the original finding dropped substantially if the contradictory evidence was published in the same journal (*M* = 50.3, *SD* = 20.8), and dropped even more if it was acknowledged by the original authors (*M* = 41.6, *SD* = 28.1). Finally, belief dropped to the lowest level (*M* = 16.4, *SD* = 18.0) when the contradictory evidence was both published in the same journal and acknowledged by the original authors. The statistical significance of the observed pattern was confirmed in a two-way within-subjects ANOVA, which showed a main effect of journal, *F*(1,95) = 347.37, *p*<.001, as well as a main effect of original authors’ reaction, *F*(1,95) = 262.09, *p*<.001, and no significant interaction, *F*(1, 95) = 1.23, *p* = .27.

## Study 2

While the results of Study 1 strongly support our predictions, it is possible that participants were overly influenced by the fact that the study was published in a lower status journal. For reasons outlined earlier, it is typically the case that a failure to replicate will be published in a lower status journal if it is published at all. But we wanted to rule out that the Study 1 findings were driven by the status language. Thus, we conducted a second study that closely replicated the first with a notable exception. In particular, rather than presenting participants with the relative status of the journals, we asked them to infer it.

### Participants

One hundred and eighty-two American users of AMT (32% female; mean age 32 years), of varied educational background, completed a survey for a compensation of $.050.

### Method

Participants were told to consider the same scenario as in Study 1, with the following two changes: First, instead of “a prestigious journal”, the original finding was said to have been published in a journal called “American Journal of Behavioral Sciences.” Second, instead “unknown researchers”, the contradictory claim was now made by “other researchers.” We thus removed any indication of journal or authorial status from the scenarios.

Like Study 1, the second study used a 2-by-2 within subjects design to manipulate how a journal editor and the authors of the original finding responded to the contradictory evidence. First, they were told that the report was published in *the same journal* “since it is customary to publish follow-up research on a given topic in the same journal as the original finding”, or that it was published in *a different journal* (‘The Journal of Social and Behavioral Science’) and the “editor of the American Journal of Behavioral Sciences rejected the paper.” The order of these two scenarios was counterbalanced across participants. Thereafter, we again presented them with the same two editorial outcome scenarios, with the addition that the original researchers acknowledged the new report invalidated their original finding. For each of the four versions of the scenario, participants estimated the validity of the original research findings, as in Study 1.

Finally, participants were told that “Academic journals differ in their status and prestige” and then asked which journal they would guess is the higher ranked journal: ‘American Journal of Behavioral Sciences’ (where the original finding was published) or ‘The Journal of Social and Behavioral Science’ (where the contradictory evidence was published in those scenarios in which it was rejected by the previous journal).

### Results

**Figure 2:**
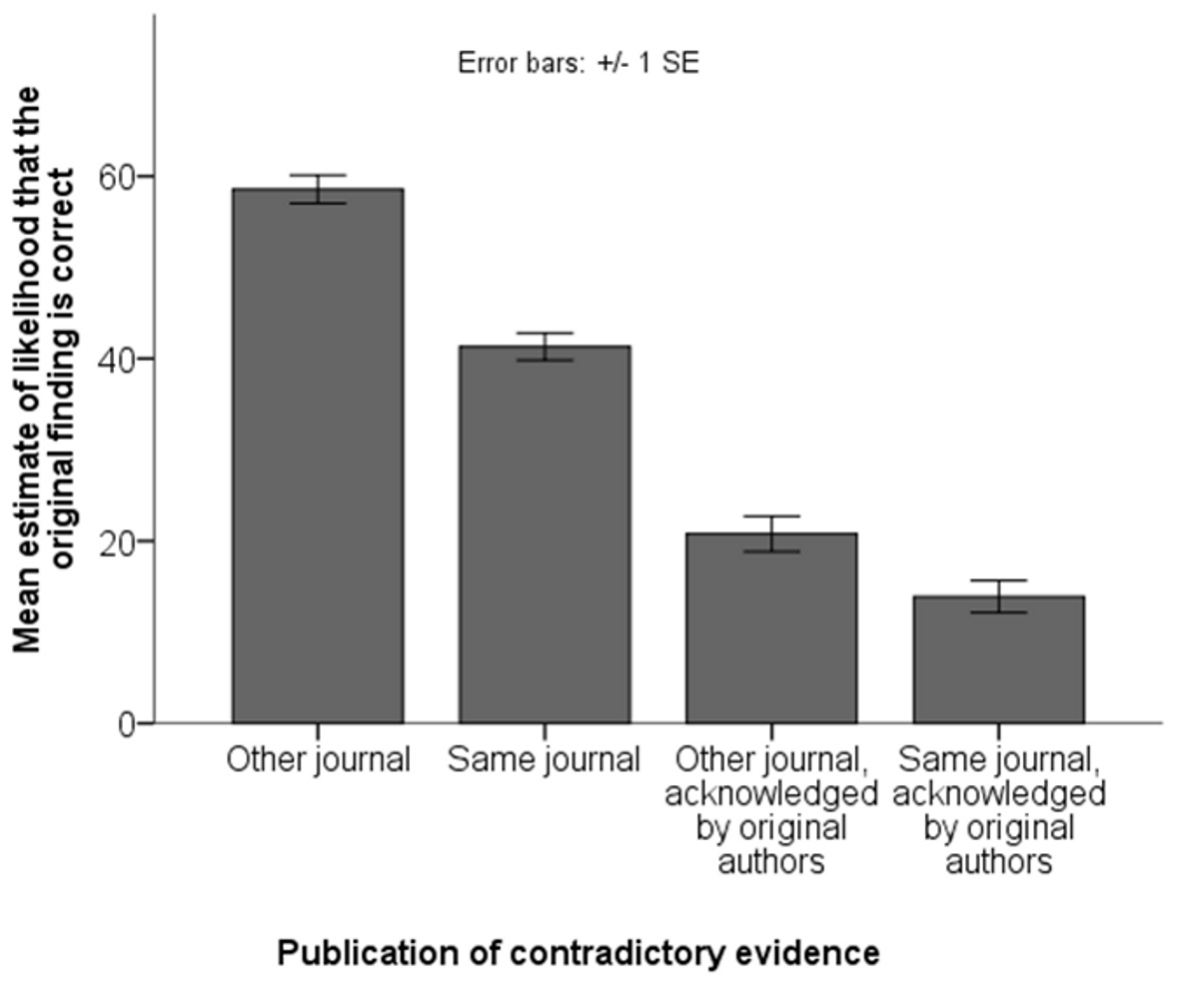
Results from Study 2.

Figure 2 shows that in case the contradictory evidence was neither published in the same journal, nor acknowledged by the original authors, belief in the original finding was still rather high (*M* = 58.6, *SD* = 20.8). Belief in the original finding dropped substantially if the contradictory evidence was published in the same journal (*M* = 41.3, *SD* = 20.0), and dropped even more if it was acknowledged by the original authors (*M* = 20.8, *SD* = 26.1). Finally, belief dropped further (*M* = 13.9, *SD* = 23.8) when the contradictory evidence was both published in the same journal and acknowledged by the original authors. A two-way within-subjects ANOVA confirmed a main effect of journal’s support, *F*(1,181) = 108.77, *p*<.001, as well as a main effect of original authors’ support, *F*(1,181) = 324.70, *p*<.001, and a significant interaction, *F*(1,181) = 18.30, *p*<.001. This interaction may be attributable to a floor effect; 35% of participants had already reached the floor of zero in their estimations of the third scenario.

Finally, among the 162 participants who indicated which journal was probably the higher ranked one, the great majority answered the journal in which the original finding was published (71%) rather than the other journal (29%), *p*<.001, binomial test. This is important because it shows that participants inferred the relative status of ostensible journals based on what typically happens to replication studies in social psychology.

## Study 3

Our final study addressed three potential weaknesses of Studies 1 and 2. First, the previous studies used a within-subjects manipulation of where the replication was published and thus risked priming participants’ responses. In the third study, we instead manipulated journal using a between–subjects design. Second, Studies 1 and 2 asked respondents to make hypothetical assessments of validity without any details of the studies. We wanted to test whether the same effect of editorial decision holds when people evaluate an actual research finding. (Our case was based on an actual study [19] and a critical failed replication [20]. See also the subsequent debate in the same journal [21], [22]. ) Finally, participants in the first two studies were recruited with no special attention to their qualification. It is possible that those who hold academic degrees in social science would be more concerned about the validity of social science findings. Or perhaps they would be more immune to the allure of journal prestige or less apt to consider the actions of journal editors in their evaluations of the validity of research findings. Finally, we also addressed whether having basic methodological details of the original and replication studies impacted evaluations.

### Participants

Seventy-three participants (44% female; mean age 36 years), all with at least a Master’s degree in a social science, were recruited among AMT users for a compensation of $1.

### Method

Participants first read the main research findings of an original publication.

A couple of years ago a study was conducted, which can be summarized as follows: *A nationally representative sample of Americans first estimated the current distribution of wealth in the United States, then constructed distributions with their ideal level of inequality. Finding: Americans dramatically underestimate the current level of wealth inequality in United States. Moreover, their ideal wealth distributions are nearly egalitarian.* This study, by researchers of distinction in the field, was published in one of the most prestigious journals in the field.

Thereafter, participants gave subjective estimates of the likelihood that the finding is valid (we refer to this as their “first rating”). Thereafter, they were informed of the outcome of a critical failed replication study. Depending on condition, participants were either told that the replication was published in the same journal or a lower-ranked journal. More specifically, after a one sentence description (in general terms) of the basic procedures and results of the failure to replicate, participants were told:

*The (replication) researchers argued that their measure had higher validity than the measure used in the previous study and concluded that the findings of [the original] study were invalid.* The replication paper was published in [the same prestigious journal as the first paper/a relatively lower-ranked online journal (not in the same prestigious journal as the first paper)].

Participants gave a new subjective estimate of the likelihood that the original finding is valid (“second rating”). Finally, participants were given a detailed summary of the specific methods and results of both the original study and the replication study (available upon request), noting that “this may of course affect your evaluation of the credibility of their findings.” After reading this information, participants made a new subjective estimate of the likelihood that the original finding is valid (“third rating”).

Summing up, each respondent’s belief in the original finding was measured three times as new pieces of information were sequentially given. The differences between subsequent measures (i.e., between the “first” and “second” ratings, and between the “second” and “third” ratings) measure the effect of each new piece of information on the respondent’s belief in the original finding.

### Results

Initial belief in the original finding was high (“first rating”: *M* = 73.6, *SD* = 21.6). We first address how simply noting the existence of a failed replication, in general terms, impacted this belief. A repeated measures ANOVA on the “first” and “second” ratings confirmed that this first piece of information led to a significant drop in belief in the original finding, *F*(1, 71) = 46.06, *p<*.001. As expected, this effect was moderated by condition, *F*(1, 71) = 7.82, *p* = .007, such that the mean *drop* in belief was greater when the failed replication was published in the same journal (mean drop* = *20.9, *SD* = 21.9) than in a lower-rank journal (mean drop* = *8.7, *SD* = 14.9). This finding is consistent with results from Studies 1 and 2.

We then analyzed the effect of the second piece of new information, that is, the details about the specific methods used and results obtained in the original study and the failed replication. A repeated measures ANOVA on the “second” and “third” ratings confirmed that this new information led to an additional drop in belief in the original finding, *F*(1, 71) = 15.81, *p<*.001. Moreover, the size of this effect did not depend on condition, *F*(1, 71) = 0.08, *p* = .78. That is, the mean drop in belief was similar whether the failed replication had been published in the same journal (mean drop* = *10.9, *SD* = 21.6) or a lower-rank journal (mean drop* = *14.2, *SD* = 31.0).

## Discussion

Owing partly to widely publicized doubts about the extent to which the discipline’s knowledge is real and replicable, calls for more replication have become increasingly prominent in social psychology. These calls have been answered by ambitious efforts to replicate a broad range of the social psychological findings (see, e.g., http://openscienceframework.org). Such efforts are critical to a healthy discipline. But as others have noted [9], [10], claims often persist in the minds of the scientific community or the broader public, even after they have been invalidated by failed replication attempts or further empirical scrutiny.

In this paper, we have addressed some key factors that increase the chances that contradictory evidence, especially in the form of critical failures to replicate, supplant acceptance of previously published findings. We focused on the role of two central players in the scientific community: journal editors and the authors of the original (invalidated) findings. The results of three studies support our claims about the roles of each of these players.

First, findings from our first two studies highlight the importance of how authors of original (invalidated) findings react. We found that when authors of the original findings acknowledged that new data invalidated their findings, belief in their original conclusions dropped sharply. While not a surprising finding, we think it is an important one: At an objective level, researchers are in the best position to point to any findings that invalidate their prior research conclusions. But a cursory search for how researchers typically respond to others’ failure to replicate suggests that a balanced assessment of the evidence is not the norm. Instead, our search turned up mostly combative responses, in which the original authors accused the replication researchers of not following the right procedures, or lacking the background knowledge or skillset to properly conduct a replication. Such responses are sometimes valid. But we worry that defensive postures all too often stem from a reluctance to admit we were wrong. This is unfortunate, as one journal editor noted [23, p.59]: “Scientists should not feel attacked when other scientists report failures to replicate our work; it’s not an accusation that we did something wrong. Rather, we should see failures to replicate–and successful replications–first as compliments, because people thought our work was worth paying attention to and spending time on, and second as providing more pieces to the puzzle that is the field of psychology.” It would be naïve to think we could turn this culture of reputation guarding on its head overnight. But we hope that the resurgence of interest in replication will create better conditions for not only celebrating when we were right but also for appreciating when others have shown that we were wrong.

Perhaps more important are our findings about how editorial decisions impact continued belief in discredited research findings. Across all three studies, we found that sustained belief depended heavily on whether the replication study was published in the same or different journal as the original finding. In addition, the third study demonstrated the importance of actually reading the contradictory evidence rather than just learning about its existence. It goes without saying that readers of journals in which the original paper appeared are more likely to read contradictory evidence if it is published in the same journal. These findings are particularly important when considered in light of the fact that many top journals in social psychology will not publish replications as a matter of policy [1], [14]. Such policies almost certainly discourage scholars from conducting replication studies. But our findings suggest a second important problem: editorial decisions not to publish failures to replicate likely sustain confidence in and acceptance of the original, invalidated, findings. From a scientific standpoint, this is grave problem that deserves attention. In particular, our findings suggest that establishing new journals aimed at publishing replications is not an adequate solution. Rather, it is important that the journals that publish original research also publish any contradictory evidence that goes through a fair peer-review process. Policies not to publish replications are not in the interest of science.

One solution is for journals to subscribe to a code of conduct for journal editors like that published by the *Committee on Publication Ethics* (see www.publicationethics.org). Most important for our current purposes is the code’s standard of “Encouraging debate,” which states that “i) Editors should encourage and be willing to consider cogent criticisms of work published in their journal.; ii) Authors of criticised material should be given the opportunity to respond; and iii) Studies reporting negative results should not be excluded.” The code of conduct suggests a best practice for editors of being open to publishing research that challenges or contradicts research previously published in the journal. A policy of *not* publishing failures to replicate is clearly antithetical to this code of conduct. But, from a scientific perspective, it is critical that replication efforts be given full consideration by the journal that published the original finding.

In this paper we have focused mainly on social psychology, partly due to our greater familiarity with the field. The primary reason for this focus, however, is that issues surrounding the validity of social psychological findings and the necessity of replication are the subject of intense debate within the social psychology community. However, other disciplines face many of the same problems [24], [25] and we know of no reason that our conclusions would not apply to these other sciences. Thus, one objective for future research might be to assess the robustness of our conclusions for research findings from other fields.

Perhaps a more important goal for future research is to address the boundary conditions of our findings. For instance, we kept to a minimum the details of the methods and findings we presented participants in Study 3. Furthermore, it is unlikely that participants knew about the particular case on which Study 3 was based, let alone held any strongly beliefs about it. But we might expect that persistence in belief in research findings will depend on a range of factors, including the nature of those findings and the (failed) replication, how long the original finding has been accepted as true and by how many people, and so on. A better understanding of the factors that lead invalid findings to “stick” even when the evidence contradicts them could only benefit scientific progress.
